# Working in Pairs: The Dialogic Distribution of Clinical Responsibility

**DOI:** 10.1007/s11013-026-10017-0

**Published:** 2026-09-03

**Authors:** Lauren Cubellis

**Affiliations:** Max Planck Institute for Social Anthropology, Halle, Germany

**Keywords:** Psychiatric crisis, Care, Dialogism, Responsibility, Germany

## Abstract

At an innovative site of psychiatric crisis care work, dialogically trained clinicians in Berlin, Germany transformed the skills learned from the Open Dialogue approach into a means for building solidarity in the face of precarious health structures. Learning to sit with unknowing and tolerate uncertainty, at first developed as techniques to support clients in crisis, became a way by which these clinicians navigated a shifting and unsteady health insurance landscape. In the face of budgetary restrictions and increased oversight by health insurance companies, which challenged the terms of their work and ethical commitments, they supported each other by reconfiguring the terms of clinical responsibility: they dispersed the authoritative gaze most often cultivated in institutional psychiatry and risk management approaches, and developed a means for attending to the uncertainty of crisis by sharing the burden of unknowing with each other. They did this through their unwavering commitment to working in pairs.

## Introduction

### Insurance Strategies

I was tucked into a corner with Adeline,^1^ one of my interlocutors on *Team Nord,* as people slowly filled the large meeting room. Carrying coffee or tea from the tables outside, greeting colleagues and getting comfortable, the collected staff of the Berlin psychiatric crisis care teams were gathering for the biannual *Groß Team Tag,* or big team day. Twice a year, the multiple teams of this social service organization met as a large group to discuss their work, the challenges they faced, and plans for the months ahead. These teams provided community-based psychiatric crisis care informed by the principles of Open Dialogue: an intervention grounded in a Bakhtinian approach to language that emphasized the importance of social networks and relational thinking in supporting people through crisis (Cubellis 2022; Seikkula & Arnkil, 2006; Olson et al. 2014; von Peter et al., 2023).

It was 2018, and over the last three to four years the organization had been contracting. Reduced investment from partner insurance companies meant that they were no longer able to sustain the ten teams working across Berlin’s ten different neighborhoods that had existed at the height of their project. Now, reduced to just three teams, they had seen numerous colleagues quit or be let go, and as individual clinicians they had taken on steadily increasing client loads. In the early days of the project, each clinician was the primary contact person for roughly fifteen to twenty clients. By the time of my fieldwork, a full-time clinician was working as the first contact person for close to forty clients. They might be the second point of contact for as many as thirty more. The increased workload, the departure of beloved colleagues, and the insecurity provoked by their dependence on insurance company funding, meant that, in many respects, morale was low at this particular *Groß Team Tag*, and had been for some time.

Dr. Denzer, their director, outlined the latest contingency plan: a twelve month contract with one of their insurance partners in which their primary goal would be—once again—to demonstrate cost savings through reduced hospitalization. But, in contrast to previous contracts, this proposal provided for less services (less skills and recovery groups, no use of the *Krisenpension*, or crisis respite center, less frequent meetings with clinicians over the long term) and was meant to help the “less acute” clients who had been brought on during the expansion of the project. This was one of many such contracts that limited the services the teams could provide, and undermined the comprehensiveness of their approach.

These limitations made many team members profoundly uncomfortable, as they did not think they would be able to provide “good care” under such a structure. Moreover, from a practical perspective, it meant they would have to provide different degrees of care for different clients, depending on the type of insurance contract the client belonged to. This meant holding dueling approaches to one’s work simultaneously, as well as patrolling a boundary along which clinical judgment and practical expertise must be reconfigured to match financial allocation. The reduction in services chipped away at what the teams felt actually *worked* about their approach: its comprehensiveness, its flexibility, their ability to adapt to a client’s particular needs, and, most importantly, it threatened their ability to work in pairs.

After Dr. Denzer finished his presentation, a chorus of questions and concerns erupted. Dr. Denzer answered them as best he could, trying to reassure the teams, but the mood in the room was tense. As we broke for lunch, I fell in with my colleagues from *Team Nord,* and reactions were split. Julian was upset, “This contract is not enough, one year is not enough time. We can’t work well with the clients in so little time, and because of this it won’t save money, and then what happens to us?” Adeline was somewhat more resigned, “Life is precarious and uncertain, it’s just the way it is. Change is the only constant.”

The crisis team members were frustrated that the terms of their work were being reconfigured, yet again. As Julian put it, if they were not able to do their work the way they knew it had to be done in order to be done well, what then for the quality of care they offered clients? And, in a very material sense, for the sustainability of the project and their jobs, which depended on their demonstration of cost-savings for the insurance companies? Adeline offered a different viewpoint, one which indexed the way the teams continually adapted to these conditions, and drew on the principle of “tolerating uncertainty” they had learned in their Open Dialogue training. Adeline was repositioning the uncertainty they were trained to engage with clients as a means for making the existential precarity of their own work livable. By offering Julian another perspective, she demonstrated how these teams defended the limits of their own vulnerability by thinking uncertainty together. Critical to holding this uncertainty was their ability to work in pairs, which emerges here as a commitment to solidarity grounded in the dialogic distribution of clinical responsibility.

### Open Dialogue in Berlin

Dr. Denzer directed a social service organization in Berlin, Germany that had, as one of its many projects, a small fleet of Open Dialogue-trained crisis teams.^2^ These teams, of which *Team Nord* was one, offered a wide variety of services to their clients: home visits, network meetings with family and friends, skills groups, accompaniment to appointments, and a *Krisenpension,* or crisis respite center, which provided an alternative to the hospital for people in crisis. All of these services were informed by the Open Dialogue training that every staff member attended upon joining the organization.^3^ The teams were able to provide these diverse services under the structure of *integrierte Versorgung,* or integrated care, an intervention by the German statutory health insurance in keeping with the latest social service guidelines intended to create more diverse and interconnected community-based care possibilities (Karow et al., 2022; Niecke & Michels, 2019; Pfammatter & Junghan, 2012). *Integrierte Versorgung* was also part of a broader effort to reduce psychiatric hospitalization and its associated costs (Fischer et al. 2014). While the Open Dialogue model was not explicitly part of the integrated care program at the state level, the health insurance companies had space to experiment with novel service provision (Thielscher et al., 2016), and the crisis teams were originally taken on as one such innovative program.

The German health system is broadly characterized by a longstanding ethos of social solidarity, a commitment to statutory health insurance, and the availability of hospital-based care (Altenstetter, 2003; Busse et al., 2017). At the same time, it is a system that has undergone massive historical changes and withstood tremendous political pressures. “Solidarity” as a framework has taken correspondingly sensitive forms in response to these shifts. During National Socialism, the health of the body politic and the maintenance of an ideal type were central (Cocks, 2007), while the German Democratic Republic (GDR) imagined a kind of psychodynamic socialist psychiatry that could strengthen the bonds of the collective techno-economic endeavour (Leuenberger, 2007). Today, an ethos of solidarity must negotiate the politics of a global health movement, the powerful interests of the pharmaceutical industry, and the international marketization and rationalization of healthcare (Gerlinger, 2024; Gröhe, 2017; Hinrichs, 2002).^4^

While German health insurance does not operate under a managed care system like that in the United States (see Dao & Mulligan, 2016; Lester, 2019; Rodriquez, 2014), neither is it isolated or fully protected from the globalizing market forces that would shape one (Dutton, 2021; Lamping & Rüb, 2010). Clinicians in Germany face challenges resonant with those of colleagues working in places like the US, where managed care structures and the drive for cost-effectiveness have seen the “contamination” of mental health services by fiscal imperatives, leading to moral dilemmas and clinician burn out, and the increased rationalization of psychiatry at the cost of more subjective understandings (Donald, 2001; Robins, 2001). As Kirschner and Lachicotte (2001) observe in their study of US community mental health clinicians, practices of authentic self-reflection, holding open the temporality of clinical horizons, and the willingness to tolerate the ambiguity of clinical proceedings were all challenged by the dictates of managed care accounting and service allocation. The Berlin teams encountered a similar struggle, and also found themselves engaged in modes of “resistance” and “survival strategies” in the face of these structural impositions (Kirschner & Lachicotte, 2001: 452).

At the same time, critical differences afforded the Berlin teams greater latitude in these negotiations. For example, during the time of my fieldwork, the crisis teams were funded on a capitation basis, meaning they received a lump sum for each client, which they could redistribute according to their assessment of need across the project. They also used creative staffing strategies and internal resource (re)distribution in order to protect their capacity for working in pairs. Such interventions, along with a historical attachment to experimenting with alternative and utopian possibilities for care (Mair, 2026), make the German case of dialogic practice a compelling site of improvisation and resistance to the creeping demands of cost-effectiveness in the late liberal welfare state.

When the original group that would become the dialogic crisis teams started working in 2007, they were part of a small-scale volunteer operation, with the family members of persons in crisis and some sympathetic social workers and psychiatrists working out of the *Krisenpension*. In 2009, Dr. Denzer was able to establish the project as a non-profit, and position a newly-paid staff under the umbrella of the larger social service organization. From 2009 to 2014, the project expanded: new contracts with interested insurance companies meant that additional staff could be hired, more teams could be built, and additional office space rented.

In the beginning of their work, the teams engaged a very specific set of clients: those who used the hospital very frequently, and who were flagged by their insurance providers as consuming quite a lot of (expensive) services. These service users were then contacted by their insurance providers, and, if they consented to trying this form of integrated care, were referred to the crisis teams. The teams would meet the client for an informational meeting, and to see if the program would be a good fit. If all went well, they would take on the new client, who would be assigned a pair of clinicians, a first and second point of contact.

Under ideal circumstances, the teams worked with these clients over a long-term horizon, partnering with them for three years, sometimes more, and offering regular contact, emergency response, skills and recovery groups, home visits, dialogic network meetings, and stays in the *Krisenpension*. Rooted in the foundational work of Open Dialogue in Finland, the program aimed to slow down crisis, to de-pathologize it, and to minimize the recourse to hospitalization and medication (Olson et al., 2014; Putman & Martindale, 2021; Seikkula & Olson, 2003). In Finland, the use of Open Dialogue has been so effective that service users rarely experience extended hospitals stays, and are able to maintain many of the hallmark rhythms of everyday life: going to school, holding on to jobs, remaining in contact with family and friends. The results of the longitudinal Finnish studies have been examined at regular intervals over the last thirty years and continue to show positive outcomes (Seikkula et al. 2006; Seikkula et al., 2011; Bergström et al., 2018).

The spike of interest in Open Dialogue around the world over the last twenty years has sought to replicate these outcomes (Razzaque & Wood, 2015; Rosen & Stoklosa, 2016; Thomas, 2011). And while clinicians are drawn to the more humanitarian approach to crisis care the model offers, and the more generous vision of recovery (Alanen, 2009; Chase & Mosse, 2025), for funding bodies and health systems the real draw is most often the reduction in hospitalization costs. This is the way many of the international Open Dialogue-based pilot projects get funding: the promise that psychiatric crisis care will become more cost-effective, and states and institutions will save money through clients’ reduced use of the hospital.

Unfortunately, few, if any, Open Dialogue projects have been able to replicate the Finnish outcomes (Freeman et al., 2019; Mueser, 2019). This has not dampened the commitment of international practitioners, and new data, from a randomized controlled trial in the UK (Pilling et al., 2022) and a multi-sited cross-cultural study across Europe (Pocobello et al., 2025), are eagerly anticipated. At the same time, the structural realities of the initial Finnish intervention are the aspects of the practice most difficult to replicate: the coordination of immediate response, the resources of a robust welfare state, and a local population with strong social ties are critical to the practice’s original development in the region. And Open Dialogue, as a need-adapted and socially-attuned intervention, is simultaneously resilient in its ability to adjust to different contexts and profoundly vulnerable to the impacts of rationalized and cost-effective care structures.

The Berlin teams were originally able to implement a version of Open Dialogue that, from their vantage point, made a real difference in their clients’ lives. And in the early years, they *did* demonstrate cost-savings via a measurable reduction in hospital usage over a multi-year period. When the insurance companies asked that the teams take on more clients in order to yield even more cost-savings, the logic backfired, and the teams were no longer able to demonstrate the same savings against the backdrop of a broader service user population that did not rely on the hospital as often in the first place. The insurance company partners became frustrated, now seeing the teams as an expensive program that was not delivering as promised, and thus they began to increase oversight, reduce available resources, and cut into the flexible working conditions of the team that had made their dialogic practice viable and impactful in the first place.

### Unraveling Clinical Responsibility

There is a broad acknowledgement that psychiatry and psychology, as fields of medicine, struggle to demonstrate diagnostic specificity and reliable outcomes (Hacking, 1996; Kirk and Kutchins 1994; Speyer et al., 2026; Summerfield, 2008). Extensive work within these fields, and in anthropology, as well as science and technology studies, has shown the ways psychiatric categories are socially and discursively constructed: they are historically and culturally contingent and subject to revision (Cooke, 2014; Foucault, 1964, 1973; Mosher, 1992), can be retrospectively applied and legitimized (Hacking, 1995; Kilroy-Marac, 2016; Metzel, 2010), are responsive to cultural and temporal demands (Feldman & Ticktin, 2010; Haliburton, 2004; Luhrmann & Marrow, 2016; Myers, 2015), and reflect the social concerns of the moment (Estroff, 1981; Hansen, 2019; Hejtmanek, 2015). Specificity in psychiatric diagnosis remains elusive, despite the pressure from health systems and financial partners to account for these diverse experiences in categorical and quantifiable ways (Dumes, 2020; Street, 2023). While important to keep in mind when thinking about how psychiatric institutions function and provide care, more pressing in this case are the ways by which the reality of diagnostic uncertainty has only minimally affected the textures and structures of psychiatric care itself. The authority of clinical professionals remains largely intact, and they wield great power, in terms of the opportunities and choices made available to psychiatric service users, both inside and outside of institutions (Brodwin, 2013; Cooper, 2018; Meyers, 2013).

Mental health clinicians are broadly endowed with the capacity and authority to make decisions for the recipients of their care. These decision-making processes are complex, contested, often ethically-ambiguous zones of relational work (Raikhel, 2016; Wagner, 2008). They can be direct, deceptive, or calculating (Buchbinder, 2011; Davis, 2010); they can be compromised and coopted (Rodriquez, 2014; Solimeo et al., 2016); and they can be ongoing and sensitive to revision (Posner et al., 1995). In most cases, they come with an attendant form of clinical responsibility. Sometimes this is an ethically held stance, developed and nurtured in relation to professional training and discourse, and sometimes it is a legal reality, whereby a clinician is liable for what happens to their clients, and if something harmful or dangerous were to occur under the clinician’s care (Rose, 1998). Often, clinical responsibility is both of these things, and is managed through hierarchical arrangements which determine pedagogical and clinical best practices (Good, 1994; Lock & Nguyen, 2010; Prentice, 2012, 2021). This is not to say that sites of psychiatric care are uniformly rigid. Rather, they are, like most institutional spaces, sites of continuous improvisation and negotiation, their porosity responds to the situations in which they operate, bending and refactoring rules and norms to meet day to day needs and concerns (Gershon, 2019; Quirk et al., 2006).

Into this space, Open Dialogue, and the work of *Team Nord* it inspired, proffers an important divergence; it purports to undo the hierarchy of clinical authority, repositioning the power to make decisions, or to even assess what is going on, into the hands of clients and their social networks. This is an essential and long-idealized facet of the original network meetings developed by Yrjö Alanen and his team in Finland. Drawing on need-adapted and systemic family therapy traditions, these meetings foregrounded the transparency of clinical deliberation and the cultivation of polyphony and multiple perspectives, letting clients take the lead in developing their own treatment trajectories (Alanen, 1997, 2009; Alanen et al., 1991; Seikkula et al., 2011). As Open Dialogue has evolved and spread (see Putman & Martindale, 2021; Pocobello et al., 2025), this feature has spawned a reconfiguration of clinical knowledge and its politics, of the ways it is wielded and held. It opens up the clinical process to the possibility of uncertainty, of embracing expertise through a learned stance of unknowing rather than the demonstration of diagnostic specificity.

I have heard Open Dialogue practitioners describe training as a process of “un-learning,” a stripping back and letting go of the self as previously constructed by biomedical psychiatry. This can be said of practitioners coming to Open Dialogue from all directions: psychiatrists, psychologists, peer professionals, social workers, nurses, and also of service users and family members (Holmesland et al., 2010). Practitioners describe learning to work dialogically as relinquishing previously held attachments to expert knowledge, repositioning service users and their families as the relevant experts, and engaging the therapeutic process as “the person that I am” rather than a professionally constructed self. For practitioners with formal clinical training, this dismantling of the authoritative self was often disconcerting at first, and many described struggling with the idea that they should let go of the impulse to intervene, to offer a solution, or to direct a course of treatment. Over time, they learned to trust the family network and the person at the center of concern, to listen to the way the network talked about and amongst itself, and to hold open a space in which the network could find what it needed.

From a critical position, one could argue that this appears to be an abdication of clinical responsibility, a doing nothing in the face of psychiatric crisis. But this is an over-simplification. To work this way demands the cultivation of a particular kind of listening and attunement (Cubellis, forthcoming). Open Dialogue trains clinicians to ask different kinds of questions, and asking different kinds of questions is not just a practical discursive exercise, but a temporal and corporeal one. The kind of listening Open Dialogue demands is slow; it is not efficient, it is not directive, it avoids rather than arrives at conclusions (Ong et al. 2022; Sidis et al., 2022). Questions are not intended to refine, but rather to broaden discourse (Ong & Buus, 2021; Rober, 2005). Practitioners regularly describe this learning process as transformational (Schubert et al., 2021; Waters et al. 2022). To learn to listen dialogically requires not only that they learn a new way of thinking about psychiatric care, but a fundamental reformulation of the self (Valtanen, 2019). To do this well requires the ability to work collaboratively with colleagues and a willingness to resist the pressure to control by learning to distribute clinical responsibility.

### Working in Pairs

In my work with *Team Nord*, working in pairs was cited again and again as the fundamental anchor of their work: one that both allowed them to engage with clients dialogically, and to hold the weight of the uncertainty they encountered in their structural precarity. In practice, the maintenance of working in pairs was a regular and ongoing negotiation. Once a week, teams got together for a four-hour *Team Sitzung*, or team meeting, in which they would go over organizational matters and client cases. While the *Groß Team Tag* described earlier was a biannual opportunity for the multiple teams to organize together on a larger scale, the *Team Sitzung* was the regular meeting in which individual teams managed their assigned client loads and day-to-day responsibilities. The meeting was a space to discuss particular cases that were proving difficult, sometimes due to the client-clinician relationship, or to the changing needs of the client, or to the escalation of a crisis. Clinicians would present these cases to their colleagues, sometimes using a reflecting team—a mode of reflexive questioning used in Open Dialogue network meetings when clinicians think out loud together in the presence of clients. This helped them think with renewed attention to how they were experiencing the case, and it generated alternative possible imaginings through the polyphonic efforts of the group.

The weekly *Team Sitzung* of *Team Nord* was held in the largest room of their Berlin office, with tall windows looking out onto a quiet side street. The room was often used for art and recovery groups, and the walls were decorated with paintings and collages made by their clients. I was regularly impressed during these meetings by how smoothly the process of finding appointment coverage went. Team members looking for a second clinician needed only provide the basic details of the case, note whether there were any appointments already on the calendar, and communicate that they needed someone to join them. Sometimes this conversation was about the different strengths different team members offered, and who might be a good fit. But any team member could volunteer in order that there be a second person present. It did not always depend on a particular or specifically identified set of skills. The most important thing was having the second person in the room.

Facilitating this kind of organization demanded patience, and a particular attention to the ways in which efforts to support paired work were distributed and reciprocated. The coordination of a second clinician for meetings with clients was a moment when everyone pulled out their calendars. While there might have been ongoing tensions or debates within the team, frustrations with variations in approach, or exhaustion at the thought of squeezing yet another meeting into an already busy week, this was always done, as far as I could discern, with minimal stress. The collective commitment to working in pairs persisted. As one team member, Paula, explained it to me, “Even if it means more work or more clients, it is easier to do the work together. Working together helps sustain us in our jobs, and we do not get as burned out as we would if we had to do all of the work alone.” While on the surface it might seem like more work to have two clinicians present in each session, in the experience of the team members, this actually made the overall workload more sustainable. Colleagues step up to accompany you, and you do so for your colleagues in return, generating a mutual solidarity in the face of difficult work.

Working dialogically in pairs allows for the affective and practical labor of clinical responsibility to be dispersed, and reconfigured from the over-burden of an individual authority to something held collectively, in relation. While having two clinicians present to build up polyphony is a key element of Open Dialogue broadly, in Berlin it took on greater significance. It helped the Berlin clinicians resist the weight of what Paul Brodwin (2013) has called “clinical futility,” in which clinicians must grapple with the limited scope and impact of their efforts in relation to the intractable challenges they face on the ground (see also Cubellis 2018).

In a similar discussion of clinical work in the US, E. Summerson Carr has shown how clinicians practicing motivational interviewing (MI) work to relinquish traditional feelings of clinical responsibility through a reframing of their impulse to offer direct suggestions or interventions for a client’s situation (Carr, 2021, 2023). Instead, they are trained to listen for moments of ambivalence in the client’s descriptions of their circumstances, and to use open-ended and reflexive questioning to support the client in resolving that ambivalence and choosing behavioral change. Like Open Dialogue, MI holds a stance of not-knowing, reduces the fixity of clinical assessments, and retrains the professional to listen to clients in a radically different way.

But a critical difference between MI and Open Dialogue is the polyphonic structure of the dialogic network meeting. MI involves dyadic conversations between clients and clinicians; these practitioners do not have the second clinician available as a resource for sharing the uncertainty that emerges around the limitations to clinical agency. As the emphasis on working in pairs reveals, *Team Nord* developed a novel means for repositioning feelings of clinical futility: *not* by acknowledging their individual limitations to change a client’s situation or behavior, but rather by taking the pressures and uncertainties inescapably associated with the clinical role and distributing them. The members of *Team Nord* were, very literally, sharing the load; distributing the burden and creating space where feelings of futility, uncertainty, and unknowing might be collectively held (see also Ivry & Teman, 2019).

This form of unknowing resonates with what Seema Golestaneh has described as the “unknowing of authority” (Golestaneh, 2023). In her work with Sufi religious teachers in Iran, she describes how a “good” teacher is best understood as one who engages students with ever more questions, rather than providing resolutions to questions through answers. In foregrounding this kind of unknowing, which values the unending exploration of infinite and possible interpretations, the authority of the teacher is deliberately diminished, backgrounded, and takes on a much humbler position. Golestaneh is talking about texts, and the clinicians on *Team Nord* are providing care, but the attention to the meaning of words, language, and authority is resonant. The clinicians on *Team Nord* use their training in Open Dialogue to bring an unknowing into their work, which was first engaged with clients, and then within their professional relationships, creating more space for their own uncertainty, their own missteps, and their own endurance under conditions of structural precarity.

Over the course of my fieldwork, I conducted dozens of interviews with the clinicians on *Team Nord*. When I asked them what was most important to them in their work, they all talked about working in pairs. They described the relief in not having to know—in not having to provide the solution for the client, and not being solely responsible for what happened in the session or afterwards—because their partner was there. In their explanations and descriptions, this release from individual responsibility allowed them to offer alternatives and possibilities that they would not have thought of otherwise. On one occasion, Adeline explained it to me this way, “It’s the pairs that makes it different, that makes it special. In [traditional] psychotherapy you are alone with the client, but in our case, it’s better, and broader, and more diverse, when there is someone sitting there with you who has a different idea, or who still manages to have an idea, even when you don’t. This makes it less tight, less narrow. There is not just one way to proceed, but many, many ways, and all ideas are allowed. I think this is really what makes it special, that you can think with your colleague. Not just in speaking with them but experiencing with them.”

Working in pairs, then, was a fundamental part of the way the *Team Nord* clinicians approached their jobs, and something the staff had been insistent over the years was absolutely essential to their work. As Paula explained it, “The responsibility is then on two people. There are two people, two different people, to hear what the client is saying and to react to it. Because of this I can be more relaxed, and being relaxed also somehow makes me more attentive. I can be more attentive because I can see more and hear more when there are two of us, I don’t have to worry the whole time that I will catch everything, that I will get it exactly right.” In being able to share the responsibility of attending to the client, Paula articulated being able to pay better attention. Lessening the anticipatory pressure to pay attention actually allowed for greater attention to be made available.

As reduced contracts and increased oversight by the health insurance companies demanded the renegotiation of their practice, the teams were unmovable on this point. Many of them told me that if, one day, the ability to work in pairs were to be taken away, this would be the end of their willingness to stay with the job. It was the way by which they refused the authoritative premises of psychiatric treatment-as-usual, and resisted the pressures to cost-effectiveness imposed by the health insurance companies.

### Working with Clients

During my time with *Team Nord,* I took part in Open Dialogue-style network meetings.^5^ Some of these were brief or multi-week encounters, others lasted the length of my fieldwork. I often worked with Marthe and Julian. Marthe and Julian had both done a year of Open Dialogue training upon joining the team and had been working with the project for nearly five years. Marthe was finishing her training as a depth-psychotherapist (a version of psychotherapy informed by psychoanalysis), and Julian had studied political science before training as a social worker. They drew on these additional professional orientations, as well as their personal experiences, in coordination with what they had learned in Open Dialogue.

A young woman living on her own in Berlin, had been working with Marthe and Julian for just over a year when I first met her. Liv was studying to be a concert pianist, Marthe explained, but during her training had been hospitalized twice and received a bipolar diagnosis from the hospital doctors. She had grown up in the United Kingdom but held a German passport, and her mother worked for a global NGO. Her father, from whom her mother was separated, did editorial work, and struggled with his own mental health. Her grandmother had also had a bipolar diagnosis, and committed suicide before Liv was born. Liv's family did not openly talk about her grandmother’s, her father’s, or her own health. This left her with a deep sense of foreboding.

Liv expressed a desire to avoid going to the hospital, but, at the same time, she made use of the hospital when she felt unwell. On one hand, Marthe and Julian wanted to support Liv's decision to go the hospital when that was what she felt she needed. On the other hand, they hoped to reduce her hospital use, knowing that under the increased scrutiny from the insurance companies it would be noted every time she went, and that they would have to explain why they were unable to prevent it. They grappled with this forced reconfiguration of their care work, fighting to find a space in which they could offer Liv the kind of dialogic approach they were committed to, and which would affirm Liv's agency in choosing her own treatment trajectory, while sitting with the risk her hospital use presented to both her continued care and the future of their project. Repeated hospitalization meant she might be denied an extension of her current care contract, and her hospital stays contributed to the increased rate of hospitalization for the team’s clients overall. This increased the risk of the insurance companies abandoning the project all together.

I saw Liv, with Marthe and Julian, over the course of eight months, either all together or with me filling in as the second person when one of them was off work or needed elsewhere. About four months into my time with Liv, Julian texted to tell me she had gone to the hospital. It happened on the precipice of her final piano exam. Julian gave me the information of the hospital she was in, and the time he planned to visit, and asked if I would meet him there.

The clinic was in the center of Berlin: an imposing grey-brown building with large wide windows along a dissonantly scenic stretch of canal. I found Julian outside the entrance to the clinic, and we sat down for a minute to check in before heading up to the ward. Julian had had an appointment scheduled with Liv in the weeks before her exam, but she canceled it, telling him she was too busy preparing. But she assured him she was doing well. They had another appointment scheduled for the day just before the exam, to which she did not show up. Julian started to worry. He texted her, asking her to let him know how the exam went. It was not until three days later that he heard from her, telling him she had gone to the hospital. She had appeared to be doing well when he last saw her, Julian reflected, doubt creeping in retrospectively. It was not clear to him how and when she had decided to go to the hospital, but he did know that she did not take her exam, and that her situation had gotten much worse.

Julian and I took the elevator up to the ward and were let through the locked doors to proceed down a long, pale yellow hallway. I could see the wide windows at the far end, looking out, through the grey, towards the canal below. Julian asked a tall, muscular, heavily tattooed man by the nurses’ station for Liv, and he, very friendly, pointed us to her room.

When Liv came out to meet us, it was clear that she was struggling. She seemed tired, disoriented, dehydrated, and in pain. She said things that were difficult for us to understand. Julian tried to ask her how she was, how she had been doing. She said she was ok, that she was feeling better, and that her mother who was not her mother was actually Jewish, and that earlier she couldn’t tell anyone, but now it was ok, and she wanted to tell us everything, and that her family that was not her family had this chain of stores, like that Canadian family, the Loblaw family, and that she supported Catherine Lobé for getting plastic surgery, and she was thinking about getting plastic surgery herself because the music world was so judgmental of overweight people, her piano teacher played in front of Rubenstein at Juilliard and it was unfair because she was never the skinniest and she had a hard time, but Frank Sinatra, Old Blue Eyes, and Debussy, Debussy was why she believed in God, Debussy was sunshine, but it was her other teacher who taught everyone that Schubert was not a joke, while her father was losing his mind because of his alcoholism the family was battered and bruised, boarding school, she was bullied but she apologized to the Prime Minster, she wanted to be a lady, God save The Queen.

As Liv spoke, her emotions were incredibly close to the surface, different words and persons in her narrative bringing her to the brink of tears and back again, her references slipping from present into past. We occasionally tried to ask her questions, to respond to the words she was saying, to use the Open Dialogue techniques we had been trained to use, but she didn’t seem to hear us. When we finally left, Julian made an appointment with her doctor to come back the following week.

Back downstairs in the hospital lobby, Julian and I sat down on a long, curved bench facing the giftshop. For a few moments we were both quiet. The whole situation made him feel bad, he said, and he was asking himself over and over what more he could have done, how could he have helped her before it got so bad. There was no way, I tried to tell him, she had told him she was fine, how could he have known? “The whole environment is so depressing,” he shook his head, and continued, “the bare walls, the bright lights, and she is so alone there, she has no one, her family is far away, and they can’t come. I hope it was ok that we went, I hope we helped, I hope we didn’t make it worse. At least we were there, I try…,” he trailed off, “If there is no one else at least we could visit.”

The Berlin teams faced limits in the ways they could implement Open Dialogue. As with many Open Dialogue initiatives outside of the model’s native Finland, structural realities meant that adaptations were necessary. People living outside of Finland often do not have the same access to health care, benefits, stable living conditions, or social networks or familial supports, nor is the state equally well-equipped to allocate need-adapted or smoothly integrated care services (see Pope et al., 2016; Gordon et al. 2016; Hopper et al. 2020). In Finland, Liv likely would not have ended up in the hospital, or been there for as long as she was. A social support network would have been built up around her and activated when she was feeling unsteady, the crisis team would have come to her home.

I was not sure then, and I still ask myself now, what exactly was dialogical about this meeting with Liv, other than the fact that Julian and I were there, the two of us. We tried to use techniques like repeating Liv's words back to her, and the careful questions of the reflecting team, but got no traction. Being in the clinic under those conditions made it difficult to listen for or cultivate polyphony, or to attune to anything other than a feeling of anxiety.^6^ Mostly I was just glad that Julian was there, and I was not on my own. Liv was in the hospital for almost three months. We continued to visit, when we were permitted. On a number of occasions, we were told only that she was unavailable. It got worse before it got better.

### Relief

Julian and I were back at the hospital. We could see Liv through the window of the door into the ward, standing in the yellow hallway chatting with the tattooed nurse, and Julian sighed, relieved, “She looks so much better.” She came to meet us, and Julian asked where we might be able to have a meeting. Liv suggested we go down to the cafeteria. After being given permission, she led us downstairs.

We barely had a chance to ask Liv how she was doing before she launched into an explanation of all that had happened. “This mania,” she explained, “was different than previous ones. This one was not about love, it was more about time travel.” She was Cleopatra, she was her grandmother, she was in Tudor England. She felt cleansed, she told us, and she figured a lot of things out. She was starting to feel ready to leave the hospital.

Liv paused briefly, and Julian asked for a moment for him and I to speak together, initiating a space for the reflecting team:

Julian:I am really happy to see Liv doing so well.

Lauren: I am relieved that she is doing much better.

Julian: I am also a bit concerned. I wonder why she didn’t call us? Why she told me everything was alright?

Lauren: I wonder if there is a reason for Liv not to call us? Maybe we can try to understand what that reason was, so that it is not only your responsibility?

We used the reflecting team to share out loud our relief at seeing Liv doing better, and Julian’s anxieties about why she had not asked for help sooner. Liv did not respond to this directly, which was fine. Reflecting team questions are offered as just possibilities. Most important in this particular reflection was the way Julian used it to address his feelings of inadequacy around the fact that Liv did not call him. He had been struggling with this feeling since she first went into the hospital. By situating this in a reflection, he was able to expose it, to make his own uncertainty and self-doubt transparent and available, but to do so without directly putting the weight of that vulnerability onto Liv. Rather, he shared it with me. My role in that moment, as I have come to understand it, was to hold with Julian the weight of his own limitations; to help shoulder the failures of caring structures to provide for clients, the fact that clients don’t always ask for help in the ways clinicians might wish they would, and the vulnerability inherent in his inability to do more to help Liv. In tolerating the uncertainty generated by a lack of agency *with* Julian, we could accept these limitations and relinquish the desire to control for them. We could only do that by dialogically distributing that burden of responsibility, and holding space for doubt together.

On our way out of the clinic, Julian thanked me. He realized there could be a reason Liv did not call us that we couldn’t yet understand. This helped him; he had not thought of it that way before.

### Finding Solidarity

As the working conditions imposed by the insurance companies directly threatened and contorted their professional practice, the clinicians on *Team Nord* were made vulnerable in new ways. In addition to caring for the vulnerability of their clients in crisis, the clinicians were exposed to dictates and pressures beyond their control, but which had concrete effects for how they could enact their care work in the first place, extending the tolerance of uncertainty to all corners of the crisis landscape.

When Julian shared his concerns about Liv's choice to go to the hospital with me, we see a moment in which multiple risks converge: Liv's crisis was a real threat to her well-being, to her exam, and to her professional and personal desires. Her hospitalization had severe consequences for her physical and mental health, as well as her case as quantified and reviewed by the insurance companies. Julian believed that if Liv needed to go to the hospital, it was good that she brought herself there. At the same time, he wished his efforts could have supported a different outcome. His desire to help Liv avoid hospitalization, while also not marking hospitalization as a failure, already a complex duality to hold, was further confounded by the ongoing pressures of insurance company oversight.

And so, Julian took this vulnerability, and reframed it with the one tool still available to him in that moment: he shared it with me. He shared it with me using the reflecting team during our session with Liv, and he shared it with me as we sat together on the bench outside the hospital giftshop. In hearing him, and responding to him, not trying to solve the problem or find a solution, but simply acknowledging it with him and offering an alternative perspective, I was able to lighten the load. Instead of grappling with the doubt around his capacity on his own, we considered it together. Instead of holding clinical responsibility as a singular and individual burden, we distributed it.

In their reconfiguration of the relationship between responsibility and care, Susanna Trnka and Catherine Trundle (2014) have revealed the multiple meanings inherent to ideas of responsibility, and argue that the neoliberal idea of responsibilization is just one framework. By reconsidering responsibility as multiple and relational, they offer a potent inroad to thinking about the dynamics of care in relation. They write, “Crucial…is the distinction between care and neoliberal concepts of responsibility, the latter of which…foregrounds the ‘autonomous’ individual as making his or her own ‘choices’ about how to act. In contrast, the relations of care we discuss here are constituted through the dual aspects of recognition and action by one’s commitment to the welfare of the other” (2014:142). Trnka and Trundle are discussing caring relationships, broadly defined. In the case of the clinical dynamics on *Team Nord*, the focus on care between colleagues offers a new position from which to consider the dynamics of care and responsibility. There was no need for me to take over for Julian in terms of his responsibility, no need for me to make a decision on his behalf, nor did he expect me to. It was simply the work of recognition, of the willingness to sit amid doubt and uncertainty with him, and to make sure he felt that I had heard him (see also Cubellis 2020).

How would Julian’s experience have been different if we hadn’t had that moment together to tolerate the uncertainty of Liv's experience and his responsibility? This was not about “me” in any specific sense, but rather the fact that a second colleague was there; someone with whom a trusting relationship had developed and who was willing to hold the discomfort of unknowing. The situation of the Berlin crisis teams, and the ways they worked with vulnerability and responsibility under precarious conditions, helps to illuminate what might be the necessary conditions of dialogic work. Julian and I held uncertainty together, and I saw Julian and Marthe, and their colleagues, do the same on many occasions. But the pressures from the insurance companies to circumscribe their work according to cost-effective dictates threatened this capacity in concrete ways, forcing *Team Nord* to grapple with not only the risk of failure in their project, but of failure to each other.

Working in pairs is a simple but fundamental reconfiguration of the structures of clinical responsibility and institutional power. Rather than organizing clinical decision-making according to a professional hierarchy, the presence of two clinicians, neither with the authority to dictate or unilaterally direct possibilities for care, begins to unravel the power invested in diagnostic and pathologizing structures (Gaines, 1992; Lester, 2019; Stevenson, 2012, 2014). Through the dialogic distribution of clinical responsibility, the confines of care can be loosened for clients, and the burden of knowing what to do in the face of immense existential and personal suffering is reduced for clinicians. Individually held knowledge, the stuff of more traditional modes of expertise, becomes second to shared knowledge, or even more explicitly, shared non-knowledge. The work of the Berlin teams reveals how the commitment to dialogic relations, even in the face of increasingly precarious health care landscapes, encourages the ability to hold not-knowing, to embrace a non-answer, and facilitates a resonant solidarity by which to sustain the work.

## Data Availability

No datasets were generated or analysed during the current study.

## Footnotes

1 All interlocutor names are pseudonyms.

2 I conducted ethnographic fieldwork with these teams for twenty-four months between 2015 and 2020.

3 I participated in over 350 hours of Open Dialogue training over the course of my fieldwork.

4 For a more extensive exploration of how this history bears on Open Dialogue practice in Germany see Cubellis 2026.

5 It was always made clear to clients that I was not a clinician, but rather an anthropologist and researcher who had been trained in Open Dialogue.

6 See also Cooper 2018.
